# Investigation of paeonol in dermatological diseases: an animal study review

**DOI:** 10.3389/fphar.2024.1450816

**Published:** 2024-11-11

**Authors:** Jingyi Ju, Tianyu Song, Jia Shi, Jialun Li

**Affiliations:** ^1^ Wuhan Union Hospital, Tongji Medical College, Huazhong University of Science and Technology, Wuhan, China; ^2^ Plastic Surgery, Pikeli Medical Aesthetics, Wuhan, China

**Keywords:** paeonol, mechanism, dermatology, pharmacological effects, drug delivery systems

## Abstract

Cortex Moutan is the root bark of the buttercup plant Paeonia suffruticosa Andr, of Ranunculaceae family. It has been utilized in Chinese medicine for thousands of years to treat a multitude of diseases, and traditional Chinese documents allege that it has heat-clearing, antipyretic, anti-inflammatory and detoxicating properties. Paeonol is a bioactive substance extracted from Cortex Moutan, which is considered to be one of its most effective metabolites. Recent studies have illustrated that paeonol treatment can alleviate skin damage, relieve the inflammatory response in patients with numerous dermatological conditions, and inhibit anomalous proliferation of skin tissue. Accordingly, paeonol may serve as a potential therapeutic agent for a variety of skin conditions. This review summarizes the physicochemical properties and pharmacokinetics (PK) characteristics of paeonol, and mechanisms of operation in diverse skin diseases, including dermatitis, psoriasis, pruritus, photoaging, hyperpigmentation, and hyperplasticscar. Additionally, much of the evidence is based on animal experiments. Furthermore, it explores the prospects of enhancing paeonol’s efficacy through extraction, synthesis, and formulation innovations, as well as strategies to overcome its limitations in dermatological therapy. This review aims to provide a more reliable theoretical basis for the clinical application of paeonol.

## 1 Introduction

**FIGURE 1:**
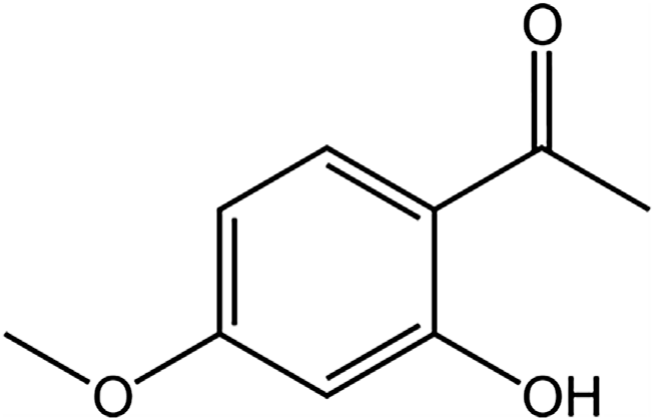
Chemical structure of paeonol (1-(2-hydroxy-4-methoxyphenyl) ethanone).

Natural plants have been an essential source of therapeutic drugs, and their intricate structural composition and extensive functional range have been the focus of relevant research (Clardy and Walsh, 2004). In recent years, with tremendous progress in the isolation, purification and characterization of natural products, pharmaceutical companies have preferred to extract bioactive substances from natural resources or to synthesize their derivatives artificially (Adki and Kulkarni, 2020). Obviously, the industry also perceives a high potential for natural products as drug precursors (Waltenberger et al., 2016). Paeonol is a bioactive phenol with the chemical formula 1-(2-hydroxy-4-methoxyphenyl) ethanone (Figure 1), isolated mainly from the root bark of the peony plant (Zhao et al., 2022). Paeonol has been demonstrated to have a wide range of biological effects, including antioxidant, anti-inflammatory, anti-tumor, and the inhibition of melanogenesis (Jin et al., 2016; Tang et al., 2020; Vellasamy et al., 2021), and these characteristics offer new approaches for developing novel drugs for the treatment of skin diseases (Li et al., 2022). In recent years, animal studies on the pharmacological functions of paeonol and its mechanism of action have been reported. Nonetheless, in the treatment of dermatological diseases, most of the current studies still concentrate on the anti-inflammatory effects of paeonol, while neglecting other biological activities. This paper reviews the mechanism of action of paeonol in skin diseases in animals and summarizes strategies to improve its low stability and aqueous solubility, which are limitations in clinical application, with a view to providing ideas for the clinical application of paeonol in skin diseases.

## 2 Search strategy and scope of literature used for this review

### 2.1 Search strategy

In this review, a comprehensive search was undertaken by three independent reviewers (JJ, TS and JS) utilizing the PubMed, Web of Science, and Scopus databases (all updated to 30 September 2023). The reviewers systematically screened the titles and abstracts to identify potentially eligible studies. The search covered publications from 2003 to 2023, focusing on studies related to effect of paeonol in the treatment of skin diseases.

Keywords and combinations of terms “paeonol”, “skin diseases”, “therapeutic effects”, “pharmacological effects”, “therapeutic potential”, “mechanism”, “skin penetration”, and “drug delivery” were used to identify relevant articles.

### 2.2 Inclusion criteria

Preclinical (*in vitro* and animal studies) was included, with a particular emphasis on peer-reviewed articles. Reviews, meta-analyses, and case reports were also considered to provide a broader perspective on the topic. Reference lists of key studies were further screened to ensure comprehensive coverage of the relevant literature.

### 2.3 Exclusion criteria

Articles not available in full text or not published in journal or those focusing on unrelated diseases were excluded.

### 2.4 Data extraction

Three researchers (JJ, TS and JS) independently extracted general characteristics (Author, Year, Disease, Agent/Dose per day, Treatment duration, Models/Animals, Main outcome). When JJ, TS and JS encountered disagreements and were unable to reach a consensus, decisions were made in consultation with a fourth reviewer, JL. In cases of incomplete data, we contacted the corresponding authors via email to request additional information.

## 3 Physicochemical properties and improvements for future research

Paeonol, 1-(2-hydroxy-4-methoxyphenyl) ethanone is a white or slightly yellow, lustrous, needle-like crystal with relative molecular mass of 166.17 and melting point of 52.5°C. It can be evaporated with steam (Adki and Kulkarni, 2020; Kim et al., 2019) in solution or in a humid environment, which underscores the importance of proper storage under a dry and sealed environment. Additionally, the phenolic hydroxyl in paeonol imparts weak acidity and certain reducibility, making it susceptible to oxidation by atmospheric oxygen and other oxidizing agents. Thus, improving the stability of paeonol is a challenge for drug application.

Regarding solubility, paeonol is very slightly soluble in water (Huang et al., 2022), with an equilibrium solubility of 0.58 mg/mL in water saturated with noctanol (Zong et al., 2016). The experimental analysis shows that the solubility and oil-water partition coefficient of paeonol in phosphate solutions of different pH ranges from 284.06 to 598.23 μg/mL and 461.97–981.17 μg/mL respectively. In terms of transdermal properties, paeonol has a small molecular mass and exhibits high permeability, with an effective passive permeability value (Pe) of 23.49 × 10^−6^ cm/s (Zong et al., 2016). Nonetheless, its hydrophobic nature hinders its passage through the stratum corneum, and the current delivery methods may limit the application potential of paeonol (Tsao et al., 2010). Therefore, increasing the transdermal delivery rate is one of the focal points of transdermal drug delivery research.

## 4 Pharmacokinetics characteristics and important implications for drug development

Rat experiments have shown that paeonol can be rapidly absorbed into the blood through the intestinal tract after oral administration and promptly distributed in many organs such as heart, brain, kidney and liver (Wu et al., 2003). The T_max_ (Time of maximum concentration) and T_1/2_ (half-life) of paeonol are relatively short. Pharmacokinetics (PK) studies manifested that after oral administration for two different doses (25 mg/kg and 50 mg/kg), the paeonol reached T_max_ at around 0.18 h and 0.19 h and was then eliminated from rat plasma after its T_1/2_, which was about 0.68 h (Hu et al., 2020). The PK profile of paeonol, while decreasing the risk of toxicity due to its accumulative properties in the body, also means that it has a rapid first-pass metabolism, which undoubtedly reduces the bioavailability of paeonol (Adki and Kulkarni, 2020). Nevertheless, the experimental results indicated that the intranasal administration of paeonol was not only rapidly and completely absorbed by rats, but also avoided the first-pass effect and the consequences caused by the blood-brain barrier (Chen et al., 2010). The C_max_ of paeonol by intranasal administration was achieved in 3 min, and it decreased rapidly from 4.0 μg/mL to 0.5 μg/mL within 30 min. In addition, the AUC (Area under the concentrationtime curve) of intranasal administration was 52.37%—about three-times greater that of oral administration, which was 15.81% (Chen et al., 2010).

Paeonol is rapidly absorbed and extensively metabolized in the body. Experiments on rats showed that after oral administration of 25 mg/kg paeonol, the highest concentration was observed in the liver at 10 min (3460.00 ± 1830.82 ng/g), followed by the heart and spleen. Additionally, paeonol is capable of crossing the blood-brain barrier to enter the brain (Hu et al., 2020). The main pathways of paeonol phase I metabolism are demethylation and hydroxylation. Nuclear magnetic resonance (NMR) and ultra-high-performance liquid chromatography-quadrupole time-of-flight mass spectrometry (UHPLC-Q-TOF-MS) analyses yielded a total of six major metabolites of paeonol, which are bound to sulfuric and glucuronic acid in phase II metabolism (Adki and Kulkarni, 2020). Based on the distribution characteristics of paeonol, it can be assumed that paeonol is mainly metabolized in the liver (Hu et al., 2020). The metabolites of paeonol have also been also detected in bile, suggesting that it maintains metabolite concentrations *in vivo* through hepatic-intestinal circulation (Hu et al., 2020).

Paeonol and its metabolites are mainly eliminated in the urine as conjugates (Hu et al., 2020). Quantitative measurements manifested that the combined urinary excretion rates of the four metabolites after 48 h were 29.75%, 26.63% and 26.52% in rats given doses of 12.5, 25 and 50 mg/kg of paeonol, respectively, which were much higher than those of 0.03%, 0.04% and 0.02% in feces (Hu et al., 2020). In addition, the absorption of paeonol is a first-order process, which is facilitated by both acidic and hypertonic conditions.

Given the rapid absorption and its short T_1/2_ of paeonol *in vivo*, optimizing routes of administration (such as intranasal administration to circumvent the first-pass metabolism), and exploring strategies to enhance bioavailability and prolong the duration of action should be considered to harness the pharmacological activity of paeonol more effectively. Furthermore, there is a necessity for assessing the risk of accumulation within the body due to paeonol’s extensive distribution across multiple organs upon entering the bloodstream. Moreover, residence time in the body and potential side effects of paeonol can be predicted by understanding its metabolic and excretion profiles.

## 5 Pharmacological effects of paeonol on each skin disease

In recent years, despite the increasing pharmacological effects of paeonol in the treatment of skin diseases, the evidence available to support its efficacy remains insufficient. This article aims to critically analyze and synthesize the pharmacological effects and underlying mechanisms of action of paeonol reported in contemporary studies on the treatment of skin diseases, with a significant number of these studies conducted in animal models. By elucidating the potential mechanisms of paeonol, we aspire to contribute valuable insights that may aid future clinical research involving human subjects and guide the therapeutic application of this compound in dermatology.

### 5.1 Dermatitis

**FIGURE 2:**
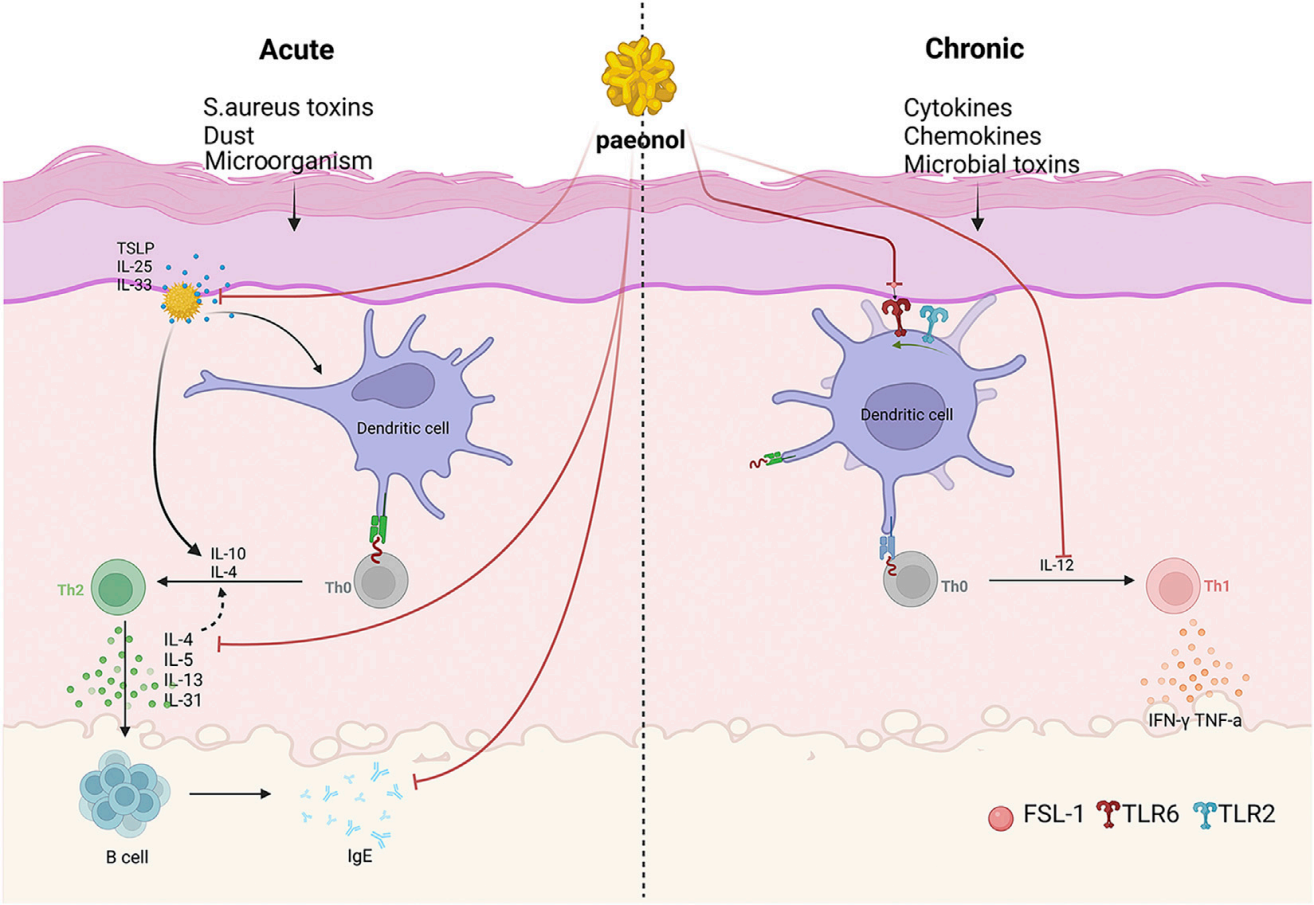
The mechanism of paeonol for inhibition of dermatitis. As shown in the figure, on the one hand, paeonol can modulate skin inflammation via inhibiting inflammatory factors and promoting anti-inflammatory factors. On the other hand, paeonol can also inhibit the inflammatory response of the body by targeting TOPK to inhibit the phosphorylation of p38 and JNKs.

Paeonol exerts anti-inflammatory and immunomodulatory effects through multiple pathways and is therefore extensively used in the treatment of various skin inflammatory conditions (Figure 2) (Hu et al., 2020). Atopic dermatitis (AD) is one of the most pervasive chronic inflammatory skin disease, which has a prevalence of 15%–30% among children and 2%–10% among adults (Zhong et al., 2009). It is usually a chronic, relapsing inflammatory skin disease, which is related to the abnormal activation of Th2 cells and accompanied by a significant increase in serum IgE levels (Huang et al., 2017). In acute phase, activated skin-resident dendritic cells (DCs) migrate to regional lymph nodes to prime and polarize naive T helper cells to Th2 phenotype. Th2 cells can induce IgE class switching in B cells, wherefore elevated IgE levels are often found in patients with AD. Furthermore, Th2 cells are recruited back to the skin and induce skin inflammation through effector cytokines IL-4/IL-5/IL-13/IL-31 and Th22 cells. In chronic phase, Th1 cells increasingly cluster, forming part of a skin infiltrate composed of Th1, Th2, and Th22 cells (Biedermann et al., 2015). It has been confirmed that paeonol efficaciously alleviated 1-chloro-2,4-dinitrobenzene (DNCB)-induced cutaneous AD-like lesions in BALB/c mice. The assay showed that the expression levels of inflammatory cytokines such as IL-4, IL-31 and TSLP were reduced in the skin of paeonol-treated mice. Furthermore, paeonol reduced serum IgE and TNF-α levels and inhibited MAPK/ERK/p38 pathway in mice, thereby inhibiting mast cell activation and attenuating allergic reactions (Meng et al., 2019). In addition, paeonol may also affect the activation and maturation of DCs by blocking the TLR2/MyD88 pathway, consequently regulating the Th1/Th2 ratio and alleviating AD symptoms (Hu et al., 2020). Moreover, animal research (Wang et al., 2023) demonstrated that paeonol-loaded liposomes in thermoreversible gels not only effectively encapsulate paeonol but also enhance its transdermal penetration. In addition, they alleviate inflammation caused by reactive oxygen species-induced (ROS-induced) oxidative stress, exert antioxidant effects, and improve skin dryness and desquamation in AD-model mice (Wang et al., 2023).

Paeonol has shown potential in the treatment of steroid-dependent dermatitis and solar dermatitis. Animal studies have demonstrated the efficacy of paeonol ointment in managing facial hormone-dependent dermatitis (Table 1). Furthermore, both *in vitro* and *in vivo* studies have indicated that paeonol may inhibit solar ultraviolet (SUV)-induced dermatitis by targeting T-LAK cell-originated protein kinase (TOPK) (Xue et al., 2017). Considering its demonstrated anti-inflammatory properties, paeonol holds promise for further exploration in the treatment of inflammatory skin conditions.

**TABLE 1:** The efficacy of paeonol ointment in facial hormone-dependent dermatitis.

Author, Year	Disease	Agent/Dose per day	Treatment duration	Animals	Main outcome
Meng et al. (2019)	1-chloro-2,4-dinitrobenzene (DNCB)-induced AD-like lesions	Groups 1 (Control): acetone and olive oil (3:1 v/v), 200 μL ad us,ext. saline solution (0.9%) i.g. 2 (Model): DNCB (1.0%), 200 μL ad us,ext DNCB (0.5%), ad us,ext with saline solution (0.9%) i.g 3 (Prednisolone): DNCB (1.0%), 200 μL ad us,ext DNCB (0.5%), ad us,ext with Pred at 10 mg/kg i.g 4 (Paeonol-high): DNCB (1.0%), 200 μL ad us,ext DNCB (0.5%), ad us,ext with paeonol at 200 mg/kg i.g 5 (Paeonol-medium): DNCB (1.0%), 200 μL ad us,ext DNCB (0.5%), ad us,ext with paeonol at 100 mg/kg i.g 6 (Paeonol-low): DNCB (1.0%), 200 μL ad us,ext DNCB (0.5%), ad us,ext with paeonol at 50 mg/kg i.g	4 weeks after adaptive feeding	6–8 weeks old female BALB/c mice	Paeonol ameliorated the morphological characteristics of DNCB-induced AD-like lesions The pharmacological effects of paeonol exhibited dose dependence
Wang et al. (2022b)	1-chloro-2,4-dinitrobenzene (DNCB)-induced AD-like lesions	Groups 1 (Control): normal saline, 200 µL on the back and 20 µL on the ear i.g 2 (Model): normal saline 200 µL on the back and 20 µL on the ear i.g 3 (Paeonol): 0.1%paeonol 200 µL on the back and 20 µL on the ear i.g 4 (Hyaluronic Acid/Paeonol): 0.1%paeonol +1 %HA 200 µL on the back and 20 µL on the ear i.g 5 (Cyclodextrin-Paeonol): 0.1%CD-paeonol 200 µL on the back and 20 µL on the ear i.g 6 (HACD-paeonol): 0.1%HACD-paeonol 200 µL on the back and 20 µL on the ear i.g	Treatment for 7 days	Male Sprague-Dawley rats weighing 200 ± 20 g and mice weighing 20 ± 2 g	All paeonol groups including paeonol, HA + paeonol, CD-paeonol and HACD-paeonol showed varying degrees of remission of skin lesions
Wu et al. (2022)	Psoriatic skin inflammation induced by imiquimod cream	Groups 1 (Normal) 2 (Vehicle): IMQ ad us,ext 3 (Dexamethasone treatment): IMQ ad us,ext with Dex (20 mg/kg) i.g 4 (Compound B_12_ L): IMQ ad us,ext with Compound B_12_ (20 mg/kg) i.g 5 (Compound B_12_ H): IMQ ad us,ext with Compound B_12_ (40 mg/kg) i.g	Treatment for 7 days	6–8-week-old female BALB/c mice	Paeonol derivative B_12_ interacted with TOPK to inhibit DNA damage-associated protein H2AX and proliferation-associated protein STAT3, effectively alleviating histological damage in IMQ-induced murine psoriasis-like skin inflammation
Ding et al. (2023)	Substance P-induced urticaria	Groups 1 (Non-sensitized control animals): saline solution (0.9%), 0.5 mL s.c 2 (SP-induced urticaria animals): SP (30 μg/mL) and Paeonol (20 mg/kg) and Cetirizine (1.5 mg/kg), 0.5 mL s.c	3 h after the injection	6–8 weeks old male C57BL/6 (wild-type [WT]) mice and BALB/c mice	Paeonol inhibited the progression of SP-induced urticaria in mice Urticaria symptoms and relevant serological indicators such as histamine, MCP-1 and TNF-α were significantly reduced in the paeonol-treated group

### 5.2 Psoriasis

Psoriasis is a chronic, immune-mediated, genetic skin disease with clinical manifestations such as pain, itching, and bleeding of the skin with abnormal proliferation of keratin-forming cells and the development of chronic inflammation (Boehncke and Schön, 2015; Parisi et al., 2015). Histopathological features of psoriasis include extensive hyperkeratosis, with horizontally confluent but vertically intermittent parakeratosis, superficial dermal edema, dilated capillaries, and perivascular lymphocytic infiltration (Balan et al., 2019). Although the pathogenesis of psoriasis has not yet been entirely elucidated, some of the mechanisms of psoriasis treatment using paeonol have been validated with some clinical success.

DCs are one of the most important specialized antigen-presenting cells in the body, capable of presenting antigenic peptides to initial T cells and thus inducing T cell activation and proliferation. Inflammatory DCs produce pro-inflammatory cytokines such as IL-23 and TNF-α, which play a key role in the onset and amplification of inflammation in psoriasis (Lowes et al., 2014). MyD88 is a typical bridging agent between TLR and IL-1 receptor family members downstream of inflammatory signaling pathways. MyD88 links interleukin-1 receptor (IL-1R) or TLR family members to IL-1R-associated kinase (IRAK) family kinases through isoform protein-protein interactions (Deguine and Barton, 2015). According to the experiment, paeonol could reduce MyD88 levels by inducing degradation or blocking synthesis, thereby obstructing TLR7-mediated signaling pathways, inhibiting DCs maturation and activation, and thus improving imiquimod-induced (IMQ-induced) psoriasis-like skin symptoms in BALB/c mice (Meng et al., 2017). *In vitro* results also demonstrated that paeonol attenuated IL-23 mRNA expression levels, thus inhibiting the maturation and activation of bone marrow-derived DCs (BMDCs), which play pro- and anti-tumorigenic roles in the tumor microenvironment and metastasis, thereby alleviating the skin damage caused by psoriasis (Vorontsova et al., 2020).

Keratinocytes produce a variety of antimicrobial peptides and cellular chemokines that interact with IL-17A and play a key role in the pathogenesis of psoriasis (Furue et al., 2020). The part of paeonol in inhibiting keratinocyte activity is receiving increasing attention. The *in vitro* experimental results demonstrated that paeonol was able to restrain the IL-17A-induced activity of human keratinocyte cell line Human Keratinocytes (HaCaT), and the mechanism might be related to the signal transducer and activator of transcription 3 (STAT3) pathway (Xie et al., 2020). Meanwhile, the results of cellular experiments illustrated that the autophagy-related 5 (ATG5) depends on the effect of paeonol on HaCaT, certifying that the ATG5 gene may be an important target for the *in vitro* treatment of psoriasis (Zhang et al., 2021).

On the other hand, Keratinocytes highly express human-derived defensin hydroxybutyrate dehydrogenase-2 (HBD-2), which promotes the chemotactic maturation of DCs and T cells. In an *in vitro* cytological model, paeonol downregulated the expression of HBD-2 gene and protein, and the mechanism may be that paeonol inhibited the activation of JNK2MAPK signaling pathway, thus alleviating the symptoms of psoriasis (Zhao et al., 2016).

In recent years, the therapeutic effects of paeonol on psoriasis have received much attention and its mechanism of action has been further elucidated. For example, *in vitro* experiments showed that angiogenesis is intimately related to the development of psoriasis, and paeonol can play a therapeutic role in psoriasis-related inflammation by modulating the VEGFR2/Akt/ERK1/2 pathway and effectively inhibiting cellular activity and lumen formation promoted by VEGF, thereby restraining angiogenesis (Luan et al., 2022). *In vivo* cell experiments confirmed that the expression level of microRNA-155 was upregulated in psoriasis mice (Xu et al., 2017). MicroRNA-155 is a class of microRNA derived from the noncoding RNA transcript of the noncoding B cell integration cluster. Its ability to both trigger inflammation and serve as a target for a variety of pro-inflammatory signals is critical to the immune response (Chen et al., 2021; Pasca et al., 2020). With the development of paeonol synthesis process, the therapeutic effects of paeonol derivatives of tannin are being gradually explored. It has been proven that the synthetic paeonol derivative 1–(5-acetyl-4-hydroxy-2-methoxyphenyl)-3-(3-(trifluoromethyl) phenyl) urea (B_12_) successfully alleviated psoriasis-like symptoms in IMQ-induced mice and significantly reduced the activity of related proteins in skin tissues. The mechanism was associated with the inhibition of proliferation-related protein STAT3 expression and precancerous factor proliferating cell nuclear antigen by B_12_ (Wu et al., 2022). In conclusion, paeonol exhibits promising therapeutic potential in the treatment of psoriasis. However, further research is necessary to explore its mechanisms of action and related derivatives in order to comprehensively understand its efficacy.

### 5.3 Pruritus

Pruritus is primarily characterized by itching and dry skin without primary skin breakdown and can be caused by a variety of skin diseases (Hawro et al., 2022). Paeonol has been verified to be an effective antipruritic drug with few side effects and plays a significant role in clinical practice (Kim et al., 2024), whereas the specific mechanism of its antipruritic effect has not been elucidated. Some experimental results revealed that paeonol can suppress pruritus by interacting with the immune pathway. Mice experiments confirmed that paeonol could perform antipruritic properties by curbing the activation of C-X-C motif chemokine receptor 3 (CXCR3) chemotactic astrocytes in the spinal cord (Wang et al., 2022a). Meanwhile, paeonol downmodulated IL-31 levels in the skin of mouse models for AD, while IL-31 has been implicated in inducing pruritus by transmitting pruritus to the central nervous system (Nygaard et al., 2016). Collectively, paeonol can treat pruritus through various pathways, while its mechanism still demands further investigation. Nevertheless, possible routes for paeonol to alleviate pruritus have been suggested. For example, paeonol downregulates the levels of specific cytokines in the spinal cord, such as Ccl6, Ccl9 and Ccl17, which may play a role in the production of pruritus. In addition, IL-2 and TNF-α secreted by Th1 cells have been identified as the main inflammatory mediators of pruritus, and the ability of paeonol to alleviate the hyperactive state of Th1 cells in patients may be one of its mechanisms of action in the treatment of pruritus. As the pathogenesis of pruritus continues to be uncovered, the therapeutic effects of paeonol will also become clearer.

### 5.4 Photoaging and photodermatosis

Photoaging refers to chronic skin damage caused by ultraviolet radiation, principally caused by medium-wave ultraviolet light (UVB). Its clinical manifestations include coarse wrinkles, skin laxity, capillary dilation and hyperpigmentation, etc (Lee et al., 2016). Paeonol has been shown to repair skin photoaging by multiple pathways (Table 2). MMP-1 is a matrix metalloproteinase (MMP) that specifically degrades Procollagen type I, a major component of the extracellular matrix, during photoaging, resulting in the destruction of the skin dermal structure. *In vivo* and *in vitro* experiments have demonstrated that paeonol mitigates the skin photoaging process by oppressing MMP-1 production mainly via an indirect endocytic protection mechanism mediated by the DLD/NRF2/ARE pathway (Sun Z. et al., 2018).

**TABLE 2:** Repair effect of paeonol on skin photoaging.

Author, Year	Disease	Agent/Dose per day	Treatment duration	Animals	Main outcome
Xue et al. (2017)	SUV-induced skin inflammation	Groups 1 (vehicle group): acetone for 3 h, ad us,ext 2 (SUV group): acetone for 3 h, ad us,ext +100 kJ/m^2^ SUV 3 (paeonol): 60 mg/kg paeonol in acetone for 3 h, ad us,ext +100 kJ/m^2^ SUV	24 h after SUV irradiation	6-week-old male Balb/c mice	Paeonol can inhibit SUV-induced skin inflammation by targeting TOPK *in vitro* and *in vivo*
Sun et al. (2018b)	UVB-induced photoaging	Groups 1 (normal): vehicle (propylene glycol and ethanol (7:3 [v:v])), ad us,ext 2 (control): UVB irradiation + vehicle, ad us,ext 3 (positive control): UVB irradiation +1% retinyl palmitate, ad us,ext 4 (PA 0.1%): UVB irradiation +0.1% paeonol, ad us,ext 5 (PA 1%): UVB irradiation +1% PA, ad us,ext 6 (PSR 1%): UVB irradiation +0.1% Paeonia suffruticosa Andr. Root (PSR), ad us,ext 7 (PSR 5%): UVB irradiation +5%PSR, ad us,ext	After 7 weeks of UVB exposure	7-week-old female albino hairless mice (HR-1; 20–27 g)	PSR and PA can prevent UVB-induced photoaging *in vitro* and *in vivo*

In addition to photoaging, excessive exposure to UV rays can also be responsible for skin inflammation. TOPK is a novel MAPKK-like protein kinase that is intimately involved in tumorigenesis. It is located upstream of p38 mitogen-activated protein kinase (p38) and c-Jun N-terminal kinases (JNKs) and is considered to be a potent therapeutic target for SUV-induced skin inflammation. In both *in vitro* and *in vivo* experiments, paeonol inhibited the phosphorylation of p38 and JNKs by targeting TOPK and blocked the formation of γ-H2AX, a downstream product acting as the biomarker of DNA double-strand breaks. The results suggested that paeonol could both repress the mobilization of JNKs and forestall skin inflammation caused by DNA damage (Xue et al., 2017).

The results of SUV irradiation experiments demonstrated that the synthetic paeonol derivative B_12_ was able to downregulate the SUV-induced TOPK signaling pathway. These findings suggest that paeonol derivatives may have potential in the treatment of light-induced skin conditions, however further clinical investigation is needed to confirm their therapeutic efficacy. (Wu et al., 2022).

### 5.5 Hyperpigmentation

Skin discoloration is mostly triggered by the accumulation and deposition of melanin produced by the body’s melanocytes and transported to the surface of the skin. The enzyme tyrosinase plays a crucial role in the synthesis of melanin (Zhou et al., 2021). Kinetic analysis has documented that paeonol can act as a non-competitive repressor of tyrosinase and inhibit melanin synthesis in a dose-dependent manner in human melanoma cells (Lin et al., 2019), which provides a rationale for paeonol treatment of hyperpigmentation. Animal experiments also indicated that paeonol significantly reduced tyrosinase activity and melanogen formation in mouse B16F10 melanoma cells, and alleviated UV-induced skin hyperpigmentation symptoms in brown guinea pigs (Peng et al., 2013). Even more interesting, tyrosinase catalyzes the production of melanin from substrates that require the involvement of oxygen molecules, and paeonol scavenges oxygen radicals in cells, thereby blocking melanin synthesis (Peng et al., 2013).

### 5.6 Hyperplasticscar

In cases of abnormal skin injury repair processes, aberrant tissue proliferation may occur and result in hyperplasticscar. Such symptoms often arise after the occurrence of burns, punctures, and other dermal injuries (Lee and Jang, 2018). During scar healing of the above wounds, fibroblasts proliferate and migrate to the wound site, are induced by stimulatory signals to become myofibroblasts and overproliferate, producing excess extracellular matrix and type III collagen (Bochaton-Piallat et al., 2016; Buhe et al., 2018). TGF-β1 is an essential signaling factor in the procedure of wound healing, which can operate through chemotaxis of fibroblasts and regulation of collagen production, and is an important contributor to hyperplasticscar (Wang et al., 2017). The outcomes of trials on isolated scar tissues displayed that paeonol is capable of prohibiting the growth of proliferative scar fibroblasts and dramatically downregulated the levels of ROS in them. Also, paeonol repressed the expression levels of TGF-β1, which may play an active role in the treatment of hyperplasticscar (Shi et al., 2018).

### 5.7 Others

Of course, in addition to its remarkable therapeutic effect on dermatological diseases, paeonol has also been demonstrated to have protective effects on other organ injuries (e.g., heart, liver, lungs). At the same time, oral paeonol also has antipyretic and analgesic effects and can be used to treat fever, headache, neuralgia, muscle pain and rheumatoid arthritis.

For example, paeonol can reduce elevated blood pressure and cardiovascular endothelial injury in spontaneously hypertensive rats through antioxidant, anti-apoptotic and modulation of vascular tone (Vellasamy et al., 2021), exert protective effects against IL-1β-induced rheumatoid arthritis in mice through PI3K/Akt/NF-κB signaling pathway (Zhai et al., 2017), inhibit inflammatory target proteins (ICAM-1 and VCAM-1) and certain cytokines (MIP-2, MCP-1, KC, and IL-1β) to treat cigarette smoke-induced lung inflammation and tissue damage as well as to inhibit nuclear translocation of NF-κB via the SIRT1/Nrf2/NF-κB pathway, thereby exerting anti-inflammatory effects in alcohol-induced acute liver injury in mice (Sun et al., 2018a). Although there is the lack of well-established clinical studies, paeonol significantly reduces myocardial infarction and no-reflow area, improves local myocardial perfusion and cardiac function, and shows potential cardioprotective effects (Yu et al., 2022).

In addition, paeonol has displayed a range of pharmacological effects in cancer therapy. For example, *in vitro* analysis showed that paeonol dose-dependently induced growth inhibition and arrest in two gastric cancer cell lines, MFC and SGC-7901, possibly accomplished by reducing the expression of the apoptosis regulator Bcl-2 protein (Sun et al., 2018a). Paeonol also inhibited breast cancer cell invasion by suppressing the signaling pathway of the gene encoding the Notch-1 one-way transmembrane receptor and reduced the expression level of transgelin two in MCF-7/PTX cells, thereby reversing the resistance of breast cancer cells to paclitaxel (Cai et al., 2014; Zhang et al., 2016).

Paeonol may also exert neuroprotective effects and could serve as a potential therapeutic agent for neurodegenerative diseases. In a study of nerve cell cultures, concentrations of 12.5, 25 and 50 μmol/L of paeonol were found to protect rats from neuronal damage induced by oxygen-glucose deficiency. This neuroprotective effect may be attributed to its ability to reduce intracellular calcium ion concentrations and to block neurological damage by acting on N-methyl-d-aspartate (NMDA) receptors as well as restraining toxic cascade response (Wu et al., 2008). In addition, paeonol was observed to attenuate neuronal damage in the hippocampus and temporal cortex of artificially induced senescent mice, thereby demonstrating that paeonol holds promise for the treatment of neurodegenerative diseases (Zhong et al., 2009).

In summary, paeonol exhibits a multifaceted targeting profile, influencing the functions of various organ systems. Its diverse pharmacological effects indicate a promising potential for further research and exploration in the context of therapeutic applications. As our understanding of its mechanisms of action continues to deepen, additional beneficial effects of paeonol are anticipated, thus paving the way for innovative treatment strategies in clinical practice.

## 6 Clinical rationale of paeonol

Paeonol, a metabolite extracted from Cortex Moutan, a botanical drug with a profound historical backdrop in traditional Chinese medicine, has a history of injection formulation in clinical application dating back to the 1970s. Advancements in pharmaceutical technology have facilitated the development of diversified paeonol formulations, including tablets, ointments, plasters, and injections. Paeonol is primarily administered orally and by injection to alleviate inflammatory and pain-related symptoms, such as fever, headache, neuralgia, and muscular pain (Zhang et al., 2019). It has also demonstrated promising therapeutic effects in the treatment of rheumatoid arthritis. Additionally, paeonol’s external preparations are used for treating various skin diseases such as eczema, dermatitis, itchy skin, mosquito and bedbug bites, as well as showing a certain effect on allergic rhinitis and cold prevention.

The combination of apocynin and paeonol was initially developed for the treatment of osteoarthritis (OA) in animals (Larkins, 2020) where it has been found at least as effective as meloxicam. In a case series study involving uncontrolled OA patients, 19 subjects were treated with a compound formulation consisting of two capsules taken twice daily (totaling a daily dose of 1240 mg of paeonol and 352 mg of apocynin). Notably, 82.6% of patients reported the treatment as effective, further corroborating the potential of this combination in alleviating OA symptoms (Larkins, 2020). Notably, a clinical trial of nanoemulsions (PM-NEs) loaded with paeonol and madecassoside (Lu et al., 2023) showed that the preparation promoted sensitive skin repair and anti-inflammation effects.

Paeonol has demonstrated immense potential as an adjuvant therapeutic agent in cancer treatment due to its broadspectrum antitumor properties. Numerous studies have revealed the remarkable advantages of paeonol combined with traditional chemotherapy drugs and autophagy inhibitor. *In vitro* cellular experiments have demonstrated that cancer cell proliferation is synergistically inhibited by the combination of paeonol and anticancer drugs. For instance, the anti-proliferation effect of human hepatocellular carcinoma cells HepG2 was significantly enhanced by paeonol in combination with doxorubicin (DOX), cisplatin (CDDP), and 5-fluorouracil (5-FU) (Xu et al., 2007b). Notably, low-concentration paeonol combined with CDDP exhibited a pronounced synergistic effect, particularly at 15.63 mg/L paeonol with 1.25 mg/L CDDP (Xu et al., 2007a). However, the synergistic interaction with DOX necessitated higher concentrations, manifesting only when paeonol concentrations were 31.25 and 62.5 mg/L in combination with 0.16 mg/L and 0.31 mg/L DOX, respectively (Xu et al., 2007b). Similarly, synergistic inhibition with 5-FU was concentration-dependent, only observed at specific combinations like 31.25 mg/L paeonol with 12.5 and 25 mg/L 5-FU (Xu et al., 2007b). The synergistic effect was further enhanced by using paeonol-PEG-NISVs (a PEGylated niosomes-mediated drug delivery systems), reducing drug dosage. 2–100 μg/mL 5-FU with 5 μg/mL paeonol-PEG-NISVs significantly inhibited HepG2 cells (He et al., 2016). In oesophageal cancer cells, particularly in SEG-1 cells, the toxicity of CDDP was enhanced by paeonol at a 1:1 ratio, via the mitochondria-dependent pathway (Wan et al., 2008).

Paeonol has also demonstrated a positive role in safeguarding vital organs against the toxic and side effects of radiotherapy and chemotherapy. Cardiotoxicity induced by epirubicin was mitigated by paeonol via the regulation of NF-κB activation *in vivo* (Li et al., 2012). Meanwhile, nephrotoxicity induced by CDDP was prevented by paeonol via inhibiting nitrosative stress. In the 4T1 tumor-bearing mouse model, paeonol alleviated epirubicin-induced hepatotoxicity by reducing ROS via inhibition of the PI3K/Akt/NF-kB pathway (Wu et al., 2015). Additionally, the ecdysterone-paeonol combination drug mitigated pro-inflammatory cytokines production in the treatment of radiation-induced oral mucositis in rats by inhibiting the NF-κB pathway, thereby compensating for the limitations of ecdysterone (Yang and Pan, 2019). Notably, paeonol exhibited a remarkable degree of selectivity and adaptability in anticancer applications, enhancing pro-apoptotic efficacy against cancer cells while simultaneously exerting anti-apoptotic effects in normal cells. In addition, combination treatment with paeonol and an autophagy inhibitor (3-methyladenine or hydroxychloroquine) showed significant synergetic effects on inhibiting ovarian cancer cell viability and promoting apoptosis *in vitro* and in animal experiments, without evident side effects. This mechanism may be related to Akt/mTOR-induced cytoprotective autophagy (Gao et al., 2019).

Despite the availability of some clinical studies investigating paeonol as an adjuvant therapy for osteoarthritis, immune diseases, and tumors, a search of relevant databases as of October 2023 revealed a limited number of clinical studies specifically focused on its application in skin diseases. This scarcity may be attributed to the current lack of widespread understanding of paeonol’s potential indications in dermatological conditions.

## 7 Enhancing paeonol efficacy through extraction, synthesis, and formulation innovations

### 7.1 Innovation in extraction and synthesis

**FIGURE 3:**
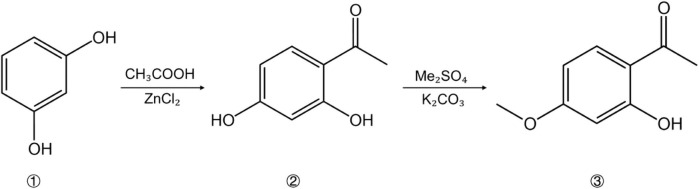
The synthetic route of paeonol: 1 M-Dihydroxybenzene; 2 2,4-Dihydroxyacetophenone; 3 Paeonol.

The traditional extraction methods of paeonol are relatively mature, including decoction, ethanol extraction, and steam distillation (Yu et al., 2007). Among them, the extraction time of decoction is difficult to determine, and it is likely to give rise to serious loss of paeonol components. Although the ethanol extraction method is simple to operate, the output rate is low. In contrast, the steam distillation not only has lower cost and higher output rate, but it is also easier to crystallize and purify. Therefore, the extraction of paeonol by steam distillation is generally preferred in the traditional process (Papandreou et al., 2002). Due to the imperfections of low output rate and difficult separation of traditional extraction methods, a series of new extraction processes have been born in recent years, including ultrasonic wave extraction, microwave-assisted extraction, enzymatic extraction, smashing extraction, supercritical fluid extraction, etc (Sun et al., 2008). In addition to their individual applications, these extraction methods can also be used in combination to further improve the extraction efficiency. For instance, the ultrasonic microwave-assisted hydrodistillation increased the recovery of paeonol to 98.49% (Chen et al., 2017). Not only that, the extraction process of paeonol is being further optimized, as evidenced by Chen et al. (2022), who improved extracting paeonol and paeoniflorin from Moutan Cortex by using deep eutectic solvents (DES) formed with choline chloride and lactic acid (molar ratio 1:2). The resulting total extraction rate of paeonol and paeoniflorin was 9.612 mg/g. Compared with organic solvents, this extraction method offers no pungent smell, is easy to biodegrade, and has great development potential. In addition, paeonol can also be synthesized by chemical methods. The conventional preparation approach is based on m-dihydroxybenzene, which undergoes an acetylation reaction and a methylation reaction to generate paeonol (Figure 3) (Yang et al., 2021). On the contrary, there are still numerous obstacles in the way of artificial synthesis, which makes it laborious to be applied in a large scale, and further research is still needed.

### 7.2 Innovations in formulation

Paeonol has been combined with various bioactive compounds, such as salidroside, asiaticoside, ozagrel, and β-asarone, to enhance its therapeutic effects or broaden its therapeutic scope. An innovative study has demonstrated the combined application of paeonol and salidroside in inhibiting melanogenesis, with the sequential release *in vitro* of these ingredients achieved through an advanced nanosphere-gel delivery system (Peng et al., 2014). In subsequent animal experiments, the rapid release of salidroside from the hydrogel promptly inhibited melanocyte proliferation, whereas the sustained release of paeonol provided continuous inhibition of tyrosinase activity in melanocytes (Peng et al., 2014). This innovative formulation not only significantly augmented the anti-melanogenesis effect but also enhanced drug stability and transdermal absorption efficiency through nanocarrier technology, pioneering a novel avenue for the treatment of pigmentary skin disorders. On the other hand, paeonol-ozagrel conjugate has shown immense potential in the treatment of ischemic stroke both *in vivo* and *in vitro* (Zhang et al., 2020). Ischemic stroke was effectively treated by synthesizing this mutual prodrug, which retained the antiplatelet and antithrombotic effects of paeonol while incorporating the anti-inflammatory and antioxidative effects of ozagrel. Concurrently, the cerebral infarction outcomes were positively influenced in animal by the combinationof β-asaron and paeonol, which regulated brain-gut axis and related signaling, thereby further broadening the application prospects of paeonol (He et al., 2017). It is worth mentioning that the combination of paeonol and glycyrrhizic acid has also been confirmed, which can effectively ameliorate the recurrent nitroglycerin-induced migraine-like phenotype in rats by regulating the GABBR2/TRPM8/PRKACA/TRPV1 pathway (Zhang et al., 2024), and provides a new drug combination idea for the treatment of migraine.

## 8 Comprehensive strategies to overcome the limitations of paeonol for its pharmaceutical use

The pharmaceutical development and application of paeonol are restricted due to its poor stability, solubility, and low bioavailability, despite showing excellent pharmacological effects and potential therapeutic value. Therefore, enhancing the stability of paeonol while enhancing its sustained release properties has emerged as a pivotal focus in pharmaceutical research. Additionally, improving the drug transdermal delivery rate is another challenge for transdermal delivery. This article aims at elucidating effective strategies to overcome the limitations of paeonol in pharmaceutical applications through modification of the drug’s chemical structure, optimization of drug delivery systems and adjustment of administration route. New approaches are expected to be developed by the implementation of these strategies for the further development and utilization of paeonol.

### 8.1 Modification of paeonol chemical structure

Numerous researchers have sought to enhance paeonol’s stability by synthesizing paeonol derivatives such as methoxy, ethoxy, piperazine, chromonylthiazolidine, phenol-phenylsulfonyl, alkyl ether, aminothiazole, tryptamine hybrids and paeononl silatie. These derivatives have undergone extensive *in vitro* bioactivity evaluations, encompassing anti-inflammatory, tyrosinase inhibitory, neuroprotective, anticancer, and antiviral activities. This topic will not be further expanded upon in this context. For comprehensive and exhaustive details, the article written by Adki and Kulkarni (2020) should be consulted.

### 8.2 Optimization of paeonol delivery systems

Innovative drug delivery systems, including tablets, hydrogels, polymeric delivery systems and lipid-based delivery systems, have been employed to address paeonol’s bioavailability and residence time, thereby potentiating its efficacy.

The gastric retention tablets of paeonol (GRT-P) enhanced the residence time of the tablet in the rat stomach by incorporating tablet accessories such as hydrophilic gel skeleton materials, hydroxypropyl methyl cellulose, foaming agent sodium bicarbonate, flow aid powder silica gel, filler lactose, and microcrystalline cellulose (Zhang et al., 2017). The study showed the retention time of GRT-P (T_max_ 2 h) was significantly extended compared to normal reference preparation (T_max_ 0.5 h).

Hydrogels are limited in terms of loading of hydrophobic drugs, significantly hindering their applications in the medical field. Therefore, various hydrogels covalently combined with cyclodextrin (CD) moieties have been synthesized by researchers to enhance their loading capacity for hydrophobic drugs. Specifically, the thermo-sensitive poly (N-isopropylacrylamide) hydrogels loaded with paeonol-β-CD complex serve as a typical example. Paeonol–β-CD complex was prepared via coprecipitation and optimized with different cross-linkers (Tsao et al., 2010), and the *in vitro* release rate was influenced by both the hydrogel composition and the release temperature. However, some issues in practical applications, such as toxic organic solvents, harmful additives, are encountered. Thus, the development of new polymer network systems was highly anticipated to break through from syneresis and low lipophilicity of hydrogel. The poly (vinyl alcohol)/silk fibroin enzymatically crosslinked semiinterpenetrating hydrogel represented an important achievement in this direction (Niu et al., 2019). Prepared through an enzymatic crosslinking method, this hydrogel achieved uniform loading of paeonol, and its release properties were dependent on the poly (vinyl alcohol)/silk fibroin ratio. Demonstrating excellent drug sustained release effects and biocompatibility, it was regarded as an ideal material for drug delivery and tissue engineering.

Polymers, owing to their large surface area, porous structures, and nanoscale dimensions, have proven to be effective as traditional drug delivery carriers (Brannon et al., 2004). The mechanisms by which their enhancement of solubility or sustained release are vary. Nanocapsules and certain dendrimers are utilized to enhance the water solubility and stability of paeonol by encapsulating it internally, thereby protecting the drug from environmental influences and allowing controlled release. The *in vitro* drug release from paeonol-loaded PLGA nanoparticles (Pae-PLGA-NPs) prepared via nanoprecipitation primarily occurs through pore diffusion on the nanoparticle surface, followed by degradation of the carrier material. This inherent property of the nanoparticles enhances the PK parameters of paeonol. Following gavage administration in rats, the AUC_(0-t)_, C_max_, MRT_(0-t)_, and T_1/2_ of the Pae-PLGA-NPs group were 3.79-fold, 1.89-fold, 1.40-fold, and 1.49-fold higher, respectively, compared to the paeonol suspension (Wang et al., 2021). Notably, intestinal absorption was also significantly improved, thereby optimizing the oral delivery of paeonol. The porous architecture of microsponge and nanospheres facilitates the permeation of release media (such as water), accelerating the dissolution of drug molecules. Additionally, drugs are released in stages due to this property, with an initial rapid dissolution of surface-bound drugs followed by a slow release of drugs from the interior pores. Furthermore, *in vitro* release profiles indicated that paeonol microsponge cream was able to show a sustained release for up to 12 h compared with paeonol cream. Moreover, dendrimers can also act by forming electrostatic/covalent bonds between drugs with terminal functional groups of dendrimers, controlling release rates through bond cleavage or alteration in physical conditions temperature and pH which are not dependent on external factors (Tripathy and Das, 2013). Dendrimers synthesized from paeonol, heterobifunctional polyethylene glycol (PEG) polymer (methoxy) and poly (amidoamine) (PAMAM) (Mignani et al., 2020) were suitable as a nasal brain transport drug delivery system due to their excellent mucoadhesive strength and biocompatibility, and direct nose-to-brain transport efficiency. Studies have shown that paeonol dendrimers exhibited maximum accumulation at 12 h after intranasal administration in mice (Xie et al., 2019).

Lipid delivery systems, which encompass microemulsions, self-micro-emulsifying drug delivery system (SMEDD), liposomes, transethosomes, ethosomes, niosomes, proniosomes, lipid-based nanoparticles, and nanoemulsion, primarily stabilize drug dispersion systems through the utilization of surfactants or phospholipids, ensuring drug stability *in vivo*, albeit with distinct structural morphologies. Microemulsions, SMEDDS, and nanoemulsions, based on emulsion principles, possess the potential to enhance drug solubility and bioavailability. Microemulsions, characterized by larger particle sizes ranging from 10 to 100 nm, are thermodynamically stable yet prone to demulsification, making them suitable for both topical and oral drug delivery. For example, microemulsions based on paeonol-menthol eutectic mixtures remarkably promoted the dermal penetration and retention of paeonol and enhance its therapeutic effect (Wang et al., 2016). In contrast, drug absorption was significantly enhanced by the spontaneous formation of droplets within the rat gastrointestinal tract, with sizes of less than 50 nm, via SMEDDS. This feature renders SMEDDS particularly advantageous for colon-targeted delivery. Furthermore, being devoid of water, SMEDDS exhibits superior long-term storage stability and offers flexibility in dosage forms. A colon-specific delivery system (Pae-SME-CSC) with paeonol loaded self-microemulsion (Pae-SMEDDS) (Zong et al., 2018) showed good localized drug release effect in the colon, exhibiting a gradual release pattern within the initial hour in artificial colonic fluid, with a release rate approaching 90% after 8 h, ultimately achieving complete release. These findings indicate that Pae-SME-CSC effectively prevents premature drug release in rat stomach and small intestine, ensuring efficient drug accumulation and release in the colon region. This resulted in a 6-fold increase *in vivo* bioavailability, enabling a therapeutic dose halved (100 mg/kg effective) for ulcerative colitis treatment, while maintaining efficacy equivalent to the traditional formulation at 200 mg/kg (Zong et al., 2018). In contrast, nanoemulsion, despite having droplet sizes comparable to microemulsions, exhibits greater structural stability, enabling their application across diverse drug administration routes. They significantly enhance drug dissolution rates and bioavailability, while also holding potential for controlled release. A novel nanoemulsion formulated with paeonol, IPM, EL35, ethanol, and water (0.0664:0.17:0.5:0.5:1.0, w/w/w/w/w) has been developed to improve the oral bioavailability of paeonol (Chen et al., 2018). After oral administration of paeonol nanoemulsion to male SD rats, the peak concentration of paeonol was significantly improved by 4-fold compared with paeonol suspension. However, there are challenges associated with the high production costs, the necessity for strictly non-toxic components, and the controlled usage of surfactants to prevent issues such as biomembrane fluidization in nanoemulsions. Furthermore, all three of them confront obstacles in terms of component selection, manufacturing processes, and cost-effectiveness, necessitating further optimization to meet clinical demands. The optimization of drug release properties is achieved through the modulation of membrane composition and structure, particularly in lipid-based delivery systems such as liposomes, transethosomes, ethosomes, niosomes. However, these systems exhibit notable differences in their composition, characteristics, and applications. The widespread application of liposomes is hindered by their susceptibility to oxidation (Li et al., 2015), despite their renowned biocompatibility and drug encapsulation capabilities. The anti-vascular endothelial growth factor (anti-VEGF) antibody-modified paeonol liposomes prepared by the film dispersion method also had the capability to delay the release of paeonol as well as to enhance its retention in the dermis (Shi et al., 2018). The skin permeability of paeonol was enhanced by the high deformability of transfersomes (Ascenso et al., 2015), yet the loading efficiency of hydrophobic drugs poses a significant challenge. In ethosomes, the skin permeability potential was further augmented by the synergistic effect of phospholipids and ethanol. Niosomes, particularly nonionic surfactant niosomes (e.g., nanovesicles), share structural similarities with liposomes but employ nonionic surfactants *in lieu* of phospholipids, thereby reducing toxicity and enhancing stability. The feature of vulnerability to clearance by the mononuclear phagocytic system limits their bioavailability. The issue of short residence time in the body has been addressed through PEG modification. A stable PEGylated niosomes-mediated drug delivery system has been characterized (He et al., 2016). In-depth exploration of the *in vivo* behavior of Pae-PEG-NISVs through rat PK experiments revealed the T_1/2_ of Pae-PEG-NISVs was 5-fold higher than that of the paeonol solution, and the AUC also showed significant differences, thereby increasing their bioavailability.

The innovative non-solvent drug loading system, the thermal-grinding supramolecular network (TGSN), which mixes raw materials including the monomer, crosslinker and drug molecules in solid state through grinding or milling, has emerged as a promising platform for targeted and controlled drug release. Its focus is on naturally derived building blocks, non-toxic additives, low-cost, and facile handling. The paeonol patch, utilizing a thioctic acid-zein-citric acid network as a carrier to deliver lipophilic paeonol, promoted the long-term release of paeonol (Chen et al., 2023). When using porcine skin from Bama miniature pig as the model skin, the cumulative transdermal release mass increased linearly during 12–96 h and the mean release rate reached up to 73.45 μg/h. Moreover, *in vivo* studies have shown that TGSN patches demonstrated superior therapeutic efficacy in treating skin eczema models in Kunming mice, outperforming traditional ointments like dexamethasone acetate (Chen et al., 2023). Further optimization of TGSN compositions and exploration of its applicability to diverse drugs could broaden its clinical applications, thereby advancing personalized medicine.

DES, as a new type of green solvent, shows unique application potential in drug delivery systems due to its significant advantages of easy preparation and no need for purification after synthesis. It can be simply obtained by heating a proper mixture of hydrogen-bond donor component and hydrogen-bond acceptor component, and the solubility and permeation ability of paeonol can be enhanced after being combined with paeonol. Specifically, research has been conducted to explore the therapeutic DES formed by paeonol with matrine (Wu and Yin, 2021), osthole (Yin et al., 2021), and lauric acid (Wu et al., 2021), respectively. These systems not only exhibit low toxicity but also significantly improve the water solubility of paeonol. Furthermore, some studies have further integrated this system with carrier systems such as microemulsion (Yin et al., 2021) or liposome (Wu et al., 2021), aiming at enhancing the stability and permeation ability of drugs. Additionally, these DES systems not only exhibit low toxicity but also significantly improve the water solubility of paeonol. New insights into the optimization of drug delivery systems are provided based on this series of innovative attempts.

### 8.3 Adjustment of administration route

Oral and intravenous administration are the most common clinical routes of administering paeonol. However, the clinical application of paeonol is limited due to its poor water solubility, as well as its low bioavailability. To enhance the bioavailability of paeonol, researchers have demonstrated significant improvements through the adjustment of administration routes, particularly through intranasal administration and inhalation administration. For instance, the paeonol-solid lipid nanoparticles-in situ gel (PaeSLNs-ISG) system combined the drug-loading advantages of SLNs with the environmental response hydrogel of ISGs (Sun et al., 2020). This combination enabled rapid gel formation of the drug within the mice nasal cavity, thereby prolonging its residence time, and it facilitated direct transport to brain regions via olfactory neural pathways, effectively bypassing the blood-brain barrier. Furthermore, research on the nasal administration of paeonol via an *in-situ* gel utilizing dendrimer nanoplatforms has demonstrated similar promising prospects, offering new possibilities for the treatment of central nervous system diseases (Mignani et al., 2020). However, despite the advantages exhibited by dendrimer nanomaterials in drug delivery, their cytotoxicity cannot be ignored, and the level of toxicity is closely related to their concentration. The cytotoxicity of PAMAM dendrimers can be effectively reduced by introducing paeonol chains *in vitro* (Mignani et al., 2020). Nevertheless, the complexity of clinical translation for intranasal administration has increased due to anatomical differences between animal models and human applications (Koo et al., 2024).

Moreover, targeted delivery of paeonol to the lungs has been achieved through the inhalation system. This method has overcome the challenges of its poor water solubility and low absorption rates associated with oral administration, resulting in a significant improvement in the absorption rate and bioavailability of paeonol. In the mouse model of acute lung injury, the C_max_ and absolute bioavailability of paeonol loaded CD metal-organic framework dry powder inhaler (Pae-CD-MOF DPI) were increased by 6.5-fold and 9.3-fold, respectively, compared with those achieved by oral administration (Li et al., 2020). However, there are some challenges in inhalation system. The flowability and aerosolization efficiency could be affected by the cubic shape of CD-MOF, necessitating optimization through surface modification. Furthermore, the residence time and absorption efficiency of the drug in the lungs might be limited by pulmonary mucosal ciliary clearance and macrophage phagocytosis. Therefore, future research is required to further optimize the particle size, shape, and surface properties of the inhalation formulation to enhance its deposition and bioavailability in the lungs (Fiegel and Gharse, 2016; Tafaghodi and Shiehzadeh, 2016).

## 9 Conclusion and future outlook

In summary, paeonol is a naturally occurring bioactive substance with promising potential in the treatment of skin diseases. The pharmacological effects of paeonol in dermatology are primarily focused on the treatment of dermatitis, psoriasis, pruritus, photodermatosis, hyperpigmentation, and hyperplasticscar, with a wide range of therapeutic mechanisms that exert their biological activity through immunomodulation, inhibition of signaling pathways, and modulation of enzyme activity. Despite the pharmacological activity of paeonol indicating great potential for clinical application in the treatment of dermatological conditions, the limited stability and low water solubility of paeonol hinder its large-scale application. Extraction and synthesis techniques for paeonol are continually being refined to enhance quality and efficacy. Currently, extraction methods for paeonol are relatively mature, however, the synthesis technology of derivatives still faces considerable technological barriers. The enhancement or expansion of paeonol’s therapeutic effects and scope may be achieved through combination with other active substances or medications. Moreover, modifications to paeonol’s chemical structure, the invention of novel drug delivery systems such as tablets, hydrogels, particles, dendrimers, and liposomes, as well as the application of nanomedicine and adjustments to routes of administration, have improved the bioavailability and residency of paeonol and enhanced the efficacy of paeonol. Nevertheless, there is still a shortage of relevant clinical applications of paeonol, and its efficacy and toxicological effects still need to be further investigated. Simultaneously, the molecular mechanisms of action in various dermatological conditions also require further elucidation.

## Glossary

AD: Atopic Dermatitis
Akt: Protein Kinase B
ARE: Antioxidant Response Element
ATG5: Autophagy-Related 5
AUC: Area under the concentration-time curves
BMDCs: Bone Marrow-Derived DCs
Ccl: C-C Motif Ligand
CD: Cyclodextrin
CDDP: Cisplatin
CXCR3: C-X-C Motif Chemokine Receptor 3
DCs: Dentritic Cells
DES: Deep eutectic solvent
DLD: Dihydrolipoamide Dehydrogenase
DOX: Doxorubicin
DNCB: 1-Chloro-2,4-Dinitrobenzene
ERK: Extracellular Regulated Protein Kinases
5-FU: 5-fluorouracil
GRT-P: Gastric retention tablets of paeonol
HaCat: Human Keratinocytes
HBD: Hydroxybutyrate Dehydrogenase
ICAM: Intercellular Cell Adhesion Molecule
Ig: Immunoglobulin
IL: Interleukin
IMQ: Imiquimod
IRAK: IL-1R-Associated Kinase
JNKs: C-Jun N-Terminal Kinase
MAPK: Mitogen-Activated Protein Kinase
MCF-7: Michigan Cancer Foundation-7
MCP: Monocyte Chemotactic Protein
MIP: Macrophage Inflammatory Protein
MMP: Metalloproteinase
Myd88: Myeloid-Differentiation-Factor88
NF-κB: Nuclear Factor Kappa-B
NMDA: N-methyl-d-aspartate
NMR: Nuclear Magnetic Resonance
Nrf2: Nuclear Factor Erythroid2-Related Factor 2
OA: Osteoarthritis
PAE-HAM-Gels: Muller-Paeonol Nanogel
PAESLNs-ISG: Paeonol-solid lipid nanoparticles-in situ gel
Pae-PLGA-NPs: Paeonol-loaded PLGA nanoparticles
PEG: Polyethylene glycol
PK: Pharmacokinetics
PTX: Paclitaxel
ROS: Reactive Oxygen Species
SIRT1: Sirtuin 1
SMEDD: Self-micro-emulsifying drug delivery system
SOCS 1: Suppressor of cytokine signaling 1
STAT3: Signal Transducer and Activator of Transcription 3
SUV: Solar Ultraviolet
TGSN: Thermal-grinding supramolecular network
Th: Helper T Cell
TLR: Toll-Like Receptor
TNF: Tumor Necrosis Factor
TOPK: T-LAK Cell-Originated Protein Kinase
TRPV1: Transient Receptor Potential Cation Channel Subfamily V Member 1
UHPLC-Q-TOF-MS: ultra-high-performance liquid chromatography-quadrupole time-of-flight mass spectrometry
UVB: Medium-Wave Ultraviolet Light
VCAM: Vascular Cell Adhesion Molecule
VEGF: Vascular Endothelial Growth Factor.
